# FABP4 in Gestational Diabetes—Association between Mothers and Offspring

**DOI:** 10.3390/jcm8030285

**Published:** 2019-02-27

**Authors:** Jolanta Patro-Małysza, Marcin Trojnar, Żaneta Kimber-Trojnar, Radzisław Mierzyński, Jacek Bartosiewicz, Jan Oleszczuk, Bożena Leszczyńska-Gorzelak

**Affiliations:** 1 Department of Obstetrics and Perinatology, Medical University of Lublin, 20-090 Lublin, Poland; zkimber@poczta.onet.pl (Ż.K.-T.); radekm1969@gmail.com (R.M.); jacek.bartosiewicz@umlub.pl (J.B.); jan.oleszczuk@umlub.pl (J.O.); b.leszczynska@umlub.pl (B.L.-G.); 2 Department of Internal Medicine, Medical University of Lublin, 20-081 Lublin, Poland; marcin.trojnar@umlub.pl

**Keywords:** gestational diabetes mellitus, fatty acid binding protein 4, neonatal anthropometry, ghrelin, leptin, adipokines

## Abstract

Fetuses exposed to gestational diabetes mellitus (GDM) have a higher risk of abnormal glucose homeostasis in later life. The molecular mechanisms of this phenomenon are still not fully understood. Fatty acid binding protein 4 (FABP4) appears to be one of the most probable candidates involved in the pathophysiology of GDM. The main aim of the study was to investigate whether umbilical cord serum FABP4 concentrations are altered in term neonates born to GDM mothers. Two groups of subjects were selected—28 healthy controls and 26 patients with GDM. FABP4, leptin, and ghrelin concentrations in the umbilical cord serum, maternal serum, and maternal urine were determined via an enzyme-linked immunosorbent assay. The umbilical cord serum FABP4 levels were higher in the GDM offspring and were directly associated with the maternal serum FABP4 and leptin levels, as well as the prepregnancy body mass index (BMI) and the BMI at and after delivery; however, they correlated negatively with birth weight and lipid parameters. In the multiple linear regression models, the umbilical cord serum FABP4 concentrations depended positively on the maternal serum FABP4 and negatively on the umbilical cord serum ghrelin levels and the high-density lipoprotein cholesterol. There are many maternal variables that can affect the level of FABP4 in the umbilical cord serum, thus, their evaluation requires further investigation.

## 1. Introduction

The association between early life insults in utero and an increased risk of developing certain noncommunicable diseases in later life has been well established [1,2,3]. Children born to mothers with obesity or gestational diabetes mellitus (GDM) are prone to develop metabolic disorders in adulthood [2,4]. Disturbances not only in the metabolism of carbohydrates but also in lipids, which have been observed in newborns of women with diabetes, may influence their metabolic profile later in life [5,6]. In women with additional metabolic stress, such as those with GDM and pre-existing obesity, there are alterations in the glucose and lipid metabolism of the adipose tissue consistent with an increased insulin resistance, leading to an increase in the circulating concentrations of fatty acids and lipids [7,8]. Several case-control studies have found that an increased insulin resistance during pregnancy is associated with abnormalities in body weight [4,7,9,10]. A higher prepregnant body mass index (BMI) and a more advanced maternal age are connected with an increased risk of GDM [11]. The molecular mechanisms by which intrauterine exposure to hyperglycemia contributes to the development of obesity and diabetes in the future life of that offspring are still awaiting explanation.

The fatty acid binding protein 4 (FABP4) belongs to a family of intracellular lipid chaperones that are expressed in active lipid metabolic tissues. This protein regulates lipid and glucose metabolism through fatty acid uptake and is involved in the transport of fatty acids, which can function as signaling molecules to the nucleus for the regulation of gene expression [12]. FABP4 is highly expressed in adipocytes, and it constitutes about 1% of all the soluble proteins in the adipose tissue [13]. Secretion of FABP4 from adipocytes is induced by lipolytic agonists or nutrient deprivation, and it is proposed that this secreted form controls the glucose production by hepatocytes and insulin secretion by the pancreatic β-cells [14,15]. FABP4 circulating levels are elevated in the serum and plasma from obese humans and mice [16,17]. Other studies have described an association between FABP4 and obesity markers, such as BMI and fat mass, as well as obesity-related diseases, including polycystic ovary syndrome [18,19]. Additionally, animal models deficient in FABP4 present reduced hyperinsulinemia and insulin resistance in obesity, which is suggestive of this adipocytokine’s effect on the interdependence between obesity and insulin resistance [20]. FABP4 appears to be one of the most probable candidates involved in the pathophysiology of GDM [21], as well as adiposity, insulin resistance, type 2 diabetes mellitus (T2DM), atherosclerosis, hypertension, coronary artery, cerebrovascular diseases, and metabolic syndrome [22,23,24,25,26].

As far as we know, no reported study has investigated the associations between the FABP4 levels in umbilical cord serum and in maternal serum and urine. In our previous study, we hypothesized that increased circulating FABP4 concentrations could persist in GDM patients after delivery and might contribute to the increased risk of T2DM and metabolic syndrome for mothers with a history of GDM [18].

We hypothesized that the FABP4 concentrations would probably be altered in the umbilical cord of term neonates born to GDM mothers. The aim of this study was also to investigate whether umbilical cord serum FABP4 levels correlate with maternal levels of FABP4 and metabolic parameters as well as with neonatal anthropometric measurements.

## 2. Experimental Section

The study comprised infants and their mothers who were in a singleton term pregnancy (after 37 weeks of gestation) and delivered at the Chair and Department of Obstetrics and Perinatology, Medical University of Lublin. The data collection was performed between March 2016 and February 2017. All the study subjects included in this study were Caucasian and they were divided into two groups. The first group consisted of 28 healthy controls, i.e., women without any metabolic disorders and with three normal results of the 2-h 75 g oral glucose tolerance test (OGTT) at 24–28 weeks of gestation. This subgroup had no concomitant diseases, received only iron supplementation, and presented proper gestational age.

The other study group consisted of 26 patients with diagnosed GDM who were on a diet and receiving insulin treatment. Of the GDM subjects, 60% were undergoing intensive insulin therapy, while 40% of them received a single basal insulin injection per day. The diagnostic criteria for GDM were based on the OGTT at 24–28 weeks of gestation: fasting glucose ≥5.1 mmol/L (92 mg/dL), a one-hour plasma glucose result of ≥10.0 mmol/L (180 mg/dL), or a two-hour plasma glucose result of ≥8.5 mmol/L (153 mg/dL) [27,28].

The exclusion criteria from the study were as follows: a multiple pregnancy, chronic infectious diseases, urinary infections, anemia, metabolic disorders (except for diagnosed GDM for the GDM group), mental illness, cancer, liver diseases, cardiovascular disorders, fetal malformation, premature membrane rupture, and intrauterine growth retardation.

Anthropometric measurements of the mothers were performed immediately before and after delivery. We defined the total gestational weight gain as the difference between the mother’s weight at delivery and her prepregnancy weight. We calculated the gestational BMI gain as well. The neonatal anthropometric measurements, including birth weight, body length, and head and chest circumferences, were performed immediately after the birth. The maternal serum levels of albumin, hemoglobin A1c (HgbA1c), and lipids were measured by a certified laboratory. The cord blood samples were taken during delivery but without any interference to the delivery. The maternal serum and urine samples were collected at the periparturient period, taking into account a 6-h fasting period. After centrifugation, all the collected cord blood serum samples, as well as the maternal serum and urine samples, were stored at −80 °C. The concentrations of FABP4 (R&D Systems, Inc., Minneapolis, MN, USA), leptin (R&D Systems, Inc., Minneapolis, MN, USA), and ghrelin (Wuhan EIAab Science Co., Wuhan, China) in these specimens were determined using commercially available kits and in compliance with each manufacturer’s instructions via a traditional enzyme-linked immunosorbent assay (ELISA). The survey was performed in duplicate for each patient.

All the patients were informed about the study protocol and detailed written consent was obtained from each patient who agreed to participate in the study. A separate information sheet was prepared for the parents of the newborns. Written signed consent from each infant’s legal guardian (mother) was also obtained.

The study protocol received the approval of the Bioethics Committee of the Medical University of Lublin (no. KE-0254/221/2015 (25 June 2015) and no. KE-0254/348/2016 (15 December 2016)).

All values were reported as the median (interquartile range 25–75%). Differences between groups were tested for significance using the Mann–Whitney *U* test. The Spearman’s coefficient test was used for the correlation analyses. The Benjamini–Hochberg correction for false positive results was performed. The multiple linear regression model was used to adjust the covariates and examine the association between the umbilical cord serum FABP4 levels and the selected biophysical and biochemical parameters of the mothers and their offspring. The regression models were adjusted for the serum FABP4 levels, the maternal and umbilical cord serum ghrelin levels, the maternal and umbilical cord serum leptin levels, high-density lipoprotein cholesterol (HDL), low-density lipoprotein cholesterol (LDL), triglycerides, and body mass index (BMI) at delivery. All analyses were performed using the Statistical Package for the Social Sciences software (version 19; SPSS Inc., Chicago, IL, USA). A *p*-value of <0.05 was considered statistically significant.

## 3. Results

The comparative characteristics of the study groups, presented in Table 1, revealed that the healthy women had a significantly lower prepregnancy BMI, BMI at delivery, and BMI after delivery. Additionally, there were decreased levels of HgbA1c, FABP4, and leptin in the maternal serum and FABP4 levels in the umbilical cord serum, as well as higher concentrations of albumin and HDL. The women in the GDM group were older and had a significantly lower gestational weight gain. No significant differences were noticed between the groups with regard to other analyzed parameters, including gestational age at delivery; the maternal serum, urine, and umbilical cord serum ghrelin levels; the umbilical cord serum leptin levels; and the neonatal anthropometric measurements (Table 1).

The Benjamini–Hochberg correction for false positive results revealed that all of the originally significant associations were still significant.

**Figure 1:**
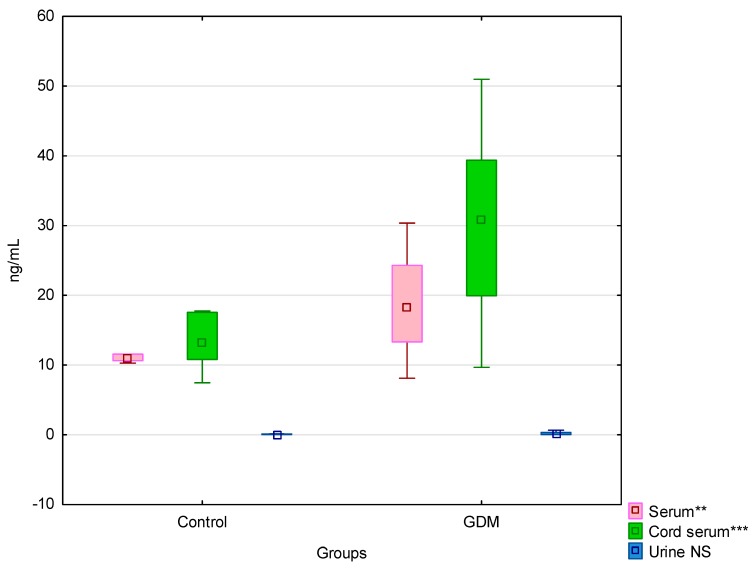
FABP4 levels in the umbilical cord serum, maternal serum, and urine in the control and GDM groups. ** *p* < 0.001; *** *p* < 0.0001; NS—not statistically significant.

We present the FABP4 levels in three materials in the control and GDM groups in Figure 1.

No significant differences in the FABP4 levels were observed in the maternal serum (*p* = 0.41), urine (*p* = 0.06), and umbilical cord serum (*p* = 0.11) between the GDM subjects undergoing intensive insulin therapy and those who were treated with a daily single insulin injection.

The umbilical cord serum FABP4 concentrations correlated positively with the prepregnancy BMI and the BMI at and after delivery. A significant positive correlation was also observed between the umbilical cord serum FABP4 levels and the FABP4 and leptin levels in the maternal serum. The FABP4 levels in the umbilical cord serum correlated negatively with the total cholesterol, HDL, LDL, and birth weight (Table 2).

In the multiple linear regression models—after adjustment for the serum FABP4 levels, the maternal and umbilical cord serum ghrelin levels, the maternal and umbilical cord serum leptin levels, HDL, LDL, triglycerides, and BMI at delivery—we noted that the umbilical cord serum FABP4 concentrations were positively dependent on the maternal serum FABP4 levels as well as negatively dependent on the umbilical cord serum ghrelin levels and HDL (Table 3).

## 4. Discussion

The study subject was FABP4—a newer adipokine which appears to be involved in the pathophysiology not only of GDM but also of adiposity, insulin resistance, T2DM, atherosclerosis, hypertension, coronary artery and cerebrovascular diseases, and metabolic syndrome [18,21,22,23,24,25,26,29,30]. In our previous study, we concluded that FABP4 seems to be an “inauspicious”/a proinflammatory adipokine [18]. We found that the maternal serum FABP4 levels were significantly the highest in the GDM patients in the early puerperium in comparison with both the controls and mothers who were characterized by excessive gestational weight gain [18].

In the current study, we assessed the concentrations of FABP4 in the umbilical cord serum of offspring of both healthy controls and GDM mothers. To the best of our knowledge, this study is the first to have evaluated associations between the FABP4 levels in umbilical cord serum and in maternal serum and urine. There is only one study, unfortunately not in English, in which the maternal and umbilical cord serum levels, as well as the FABP4 mRNA placental expression, were evaluated in patients with pre-eclampsia [31]. In the cited study, the umbilical cord blood FABP4 concentrations were significantly decreased in the early- and late-onset pre-eclampsia groups as compared to the late control group (i.e., after 34 weeks of gestation). Furthermore, the umbilical cord blood FABP4 concentration correlated negatively with the maternal serum FABP4 level and the placental FABP4 mRNA expression [31].

Our study revealed that the umbilical cord serum FABP4 levels were significantly higher in the children of the GDM mothers. However, what is also important is that the mothers with GDM, when compared with the healthy controls, presented higher FABP4 concentrations in their serum, but their urinary levels were similar. The umbilical cord serum FABP4 levels were positively associated with the maternal serum FABP4 levels. Apart from this, we performed multiple linear analyses that revealed the dependence of the maternal serum FABP4 concentrations on the levels in the umbilical cord serum. Each 1 ng/mL increase in the umbilical cord serum FABP4 concentration was linked to an increase in the maternal serum FABP4 level by 0.89 ng/mL.

Previous studies found that the serum FABP4 concentrations were significantly increased in pregnant women with GDM when compared with the controls [32,33,34]. High circulating FABP4 levels in the maternal serum of pregnant women with GDM may be explained by an increased FABP4 originating from the placenta and adipocytes [35,36]. The expression of FABP4 mRNA in the placenta and decidua of GDM patients is greater than that in normal tissues [36]. The synergistic effects of FABP4 from the placenta and adipocytes can act, via adipocytes, on the metabolic and inflammatory pathways. These activities may play crucial roles in the development of insulin resistance and T2DM [36].

### 4.1. Associations between FABP4 and Maternal Weight Parameters

Our results revealed a difference in the gestational weight gain but not in the gestational BMI gain between these two groups of mothers. We observed that the umbilical cord serum FABP4 levels positively correlated with the maternal BMI values (prepregnancy, at delivery, and after delivery) in the study subjects. These results seem to be related to the fact that the women in the GDM group, due to prepregnant overweight and obesity and with normal carbohydrate results in the first trimester, remained under strict control, so that from 24–28 weeks of pregnancy, after being diagnosed with GDM, they were successfully treated with not only a proper diet but also with insulin. Our findings can be confirmed by the previous studies that found significant correlations between the maternal FABP4 and prepregnancy BMI [34,37].

### 4.2. Associations between FABP4 and Maternal Laboratory Results

Our study revealed that lower levels of HDL were present in the GDM group. These results are consistent with the observations of other authors [9,35]. Li et al. [35] reported higher levels of BMI, gestational BMI gain, triglycerides, and FABP4 as well as lower levels of HDL in the GDM patients than in the studied controls. These findings are similar to those reported by Park et al. [38] in patients with metabolic syndrome. It is well known that women with a history of GDM exhibit altered risk factors of cardiovascular diseases, including lower HDL concentrations, when compared with mothers with healthy pregnancies [9,39,40,41,42].

We found that the umbilical cord serum FABP4 level correlated negatively with the maternal lipid profile (i.e., total cholesterol, HDL, and LDL levels). However, the multiple linear regression model performed on all the study participants revealed that the umbilical cord serum FABP4 concentrations were negatively dependent on the maternal HDL levels. This negative relationship between FABP4 and HDL seems to confirm a disadvantageous profile of this adipokine. We cannot relate our results regarding the associations between the umbilical cord serum FABP4 level and the maternal metabolic parameters to any previously conducted study results because no such results are available.

Nevertheless, we are able to relate to the accessible results of the studies concentrating on comparing GDM mothers with healthy mothers. Our previous study, performed on early postpartum women with GDM or with excessive gestational weight gain, revealed that the maternal serum FABP4 concentrations correlated positively with the lipid profile (i.e., total cholesterol, HDL, and LDL levels), and they also correlated negatively with HgbA1c concentrations in healthy puerperal mothers [18]. Yan and Wang [31], as well as Lin et al. [43], noticed that the mean maternal serum FABP4 concentrations correlated negatively with HDL and positively with triglycerides and total cholesterol in patients with pre-eclampsia. Fuseya et al. [44] and Ishimura et al. [45] showed similar correlations in healthy patients of both sexes.

### 4.3. Associations between FABP4 and Leptin

In this study, we found that leptin levels were higher in the maternal serum of the GDM patients than of the control subjects, and they were comparable between these two groups in the umbilical cord serum. The umbilical cord serum FABP4 levels positively correlated with the maternal serum leptin levels.

Several previous studies revealed direct associations between the circulating FABP4 and leptin [32,33,46,47]. These relationships can be explained by the fact that both leptin and FABP4 are mainly secreted by the adipose tissue [48]. Influenced by both prenatal maternal factors and postnatal developmental cues, the adipose tissue expansion and production of adipokines progress rapidly during postnatal life. Zachariah et al. [49] hypothesized that nonobese study participants without diabetes mellitus (DM) whose parents suffered from obesity or DM would have altered circulating adipokines compared with the individuals without a parental history of these conditions. The study showed that parental obesity was associated with significantly higher serum levels of FABP4 and leptin receptor in their offspring, whereas parental DM was linked to lower adiponectin but higher retinol binding protein 4 concentrations in the offspring. The cited authors emphasized that a parental history of DM or obesity was associated with an altered adipokine profile in nonobese, nondiabetic offspring [49]. They recommended that additional studies should be carried out to evaluate whether such preclinical biomarker alterations are able to predict the future risk of disease. This point of view is similar to ours.

Leptin and FABP4 are interactively associated with the physiology and pathophysiology of various conditions. They seem to be involved in the early placentation process [50]. On the other hand, Zhang et al. [32] showed that their interaction might cause more severe insulin resistance, since FABP4 could be involved in leptin resistance, and thereby contribute to more severe disturbances of carbohydrate metabolism in GDM mothers. Furthermore, FABP4 plays a key role in fatty acid transportation and oxidation, and it increases synergistically with leptin during the adipose inflammation process [51]. Moreover, an increased expression of FABP4 and leptin as components of the peroxisome proliferator-activated receptor (PPAR)/adipocytokine signaling pathway in the resident macrophages is typical of the atherosclerotic plaque rupture [52].

Bearing in mind our detection of positive associations between the umbilical cord serum FABP4 level and the maternal serum FABP4 and leptin levels (as well as previous results regarding the relationship between these “inauspicious”/proinflammatory adipokines and the pathogenesis of T2DM, obesity, cardiovascular diseases, and metabolic syndrome), a consideration of FABP4 and leptin in future studies of GDM offspring seems to be of the essence.

### 4.4. Associations between FABP4 and Ghrelin

In our study, the concentrations of ghrelin in the umbilical cord serum, as well as in the maternal serum and urine, were similar in both study groups. Moreover, it is interesting to point out a negative dependence of ghrelin on FABP4 in the umbilical cord serum. In our previous study, a positive correlation was found between the maternal urine FABP4 and ghrelin levels in the GDM group. However, the data on the relationship between ghrelin and FABP4 are limited. Zhang et al. demonstrated that overexpression of the ghrelin gene via the FABP4 promoter exhibits a high plasma concentration of des-acyl ghrelin, alters the development of the white adipose tissues, and improves glucose tolerance and insulin sensitivity [53].

### 4.5. Associations between FABP4 and Neonatal Anthropometric Measurements

Among the nutrients that support intrauterine growth, fatty acids are important, especially during the second half of pregnancy, when fetal growth accelerates and fat accretion increases exponentially [54]. Importantly, Makkar et al. found that perinatal hypoxia selectively upregulated the expression of FABP1, FABP3, and FABP4 and that fatty acids enhance the expression of FABP4 [54]. Coupled with the known function of FABP4 as a fatty acid chaperone, Makkar et al. hypothesized that FABP4 could be a key regulator of trophoblastic lipid transport and accumulation. Ciborowski et al. indicated that neonatal birth weight is related to the level of selected lipids and FABP4 in the mother’s blood [55]. We noted that FABP4 levels in the umbilical cord serum correlated negatively with birth weight. The offspring of our control and GDM groups had comparable anthropometric measurements, including birth weight. However, in light of previous studies, fetal metabolic programming may occur within normal birth weight ranges [56,57].

## 5. Conclusions

Our study revealed that the umbilical cord serum FABP4 levels were significantly higher in the children of the GDM mothers. In light of our study results, it seems probable that maternal condition, including maternal BMI values in the periconceptional and periparturient periods as well as maternal metabolic parameters (such as lipid profile), could have an impact on the FABP4 concentrations of the offspring at delivery. It is worth noting that the concentrations of FABP4 in the umbilical cord serum are directly dependent on the maternal serum FABP4 levels.

To conclude, there are many variables in the maternal serum that can affect the level of FABP4 in the umbilical cord serum, thus, their evaluation requires further investigation.

Although urine is an easily available and appropriable biological material, according to our findings it does not prove valuable to predict the level of FABP4 in umbilical cord serum.

## Figures and Tables

**Table 1:** Comparison of characteristics of the study subjects.

Variables	Control Group (*n* = 28)	GDM Group (*n* = 26)	*p*
**Maternal Characteristics**			
age, years	29 (24–38)	34.5 (32–41)	**0.0021** *
prepregnancy BMI, kg/m^2^	20.3 (19.5–24.4)	27.55 (24.53–29.8)	**0.000001** ***
gestational weight gain, kg	15 (11.5–15.6)	13.3 (9.2–15.0)	**0.018** *
gestational BMI gain, kg/m^2^	5.4 (3.0–5.6)	5.508 (3.11–5.72)	NS
BMI at delivery, kg/m^2^	26.3 (24.2–29.1)	32.3 (30.23–33.9)	**0.000007** ***
BMI after delivery, kg/m^2^	22 (21–23.9)	28.8 (25.3–30.65)	**0.000003** ***
gestational age at delivery, weeks	39.8 (38.8–40.6)	39.2 (37.8–39.6)	NS
**Maternal Serum**			
albumin, g/dL	3.68 (3.43–3.73)	3.46 (3.37–3.64)	**0.007** *
total cholesterol, mg/dL	249 (188–287)	209 (192.5–247.5)	NS
HDL, mg/dL	78 (75–82)	67.5 (54.5–73.5)	**0.001** *
LDL, mg/dL	129 (93–152)	107 (85.5–129)	NS
triglycerides, mg/dL	177 (150–254)	240.5 (170–261)	NS
HgbA1c, %	5.3 (4.6–5.4)	5.5 (5.2–5.6)	**0.018** *
FABP4, ng/mL	10.99 (10.63–11.56)	18.23 (13.32–24.29)	**0.00022** **
ghrelin, ng/mL	0.933 (0.646–1.115)	0.4 (0.19–1.23)	NS
leptin, ng/mL	10.43 (6.04–14.9)	18.15 (10.84–51.91)	**0.005** *
**Maternal Urine**			
FABP4, ng/mL	0.04 (0.03–0.1)	0.06 (0.02–0.34)	NS
ghrelin, ng/mL	0.102 (0.096–0.288)	0.21 (0.07–6.6)	NS
**Umbilical Cord Serum**			
FABP4, ng/mL	13.29 (10.79–17.56)	30.83 (19.94–39.38)	**0.000001** ***
ghrelin, ng/mL	0.195 (0.187–0.282)	0.32 (0.18–0.71)	NS
leptin, ng/mL	7.53 (4.9–14.01)	12.13 (4.81–27.14)	NS
**Neonatal Anthropometric Measurements**			
birth weight, g	3400 (3170–3830)	3225 (3005–3510)	NS
birth body length, cm	55 (53–56)	53 (52.5–55.5)	NS
head circumference, cm	34 (33–35)	34 (34–35)	NS
chest circumference, cm	34 (34–35)	34 (32.5–35)	NS

The results are shown as the median (interquartile range 25%–75%). Statistically significant values are given in bold. * *p* < 0.05; ** *p* < 0.001; *** *p* < 0.0001. BMI—body mass index; GDM—gestational diabetes mellitus; HDL—high-density lipoprotein cholesterol; HgbA1c—hemoglobin A1c; LDL—low-density lipoprotein cholesterol; FABP4—fatty acid binding protein 4; NS—not statistically significant.

**Table 2:** Correlation coefficient between the umbilical cord serum FABP4 levels and the clinical parameters in both study groups.

Variables	Umbilical Cord Serum FABP4	
	*r*	*p*
**Maternal Characteristics**		
prepregnancy BMI	**0.524**	**0.00026** **
gestational weight gain	−0.108	0.437
gestational BMI gain	−0.251	0.093
BMI at delivery	**0.523**	**0.00027** **
BMI after delivery	**0.574**	**0.000047** ***
gestational age at delivery	0.153	0.114
**Maternal Serum**		
albumin	−0.285	0.055
total cholesterol	**−0.522**	**0.00028** **
HDL	**−0.394**	**0.008** *
LDL	**−0.515**	**0.00035** **
triglycerides	0.005	0.97
HgbA1c	0.153	0.31
FABP4	**0.356**	**0.015** *
ghrelin	−0.279	0.060
leptin	**0.494**	**0.00047** **
**Maternal Urine**		
FABP4	−0.057	0.705
ghrelin	0.210	0.161
**Umbilical Cord Serum**		
ghrelin	−0.147	0.331
leptin	0.188	0.21
**Neonatal Anthropometric Measurements**		
birth weight	**−0.381**	**0.011** *
birth body length	−0.184	0.232
head circumference	−0.241	0.114
chest circumference	−0.216	0.159

Statistically significant values are given in bold. BMI—body mass index; HDL—high-density lipoprotein cholesterol; HgbA1c—hemoglobin A1c; LDL—low-density lipoprotein cholesterol; FABP4—fatty acid binding protein 4. * *p* < 0.05; ** *p* < 0.001; *** *p* < 0.0001.

**Table 3:** Multiple linear regression analyses for the umbilical cord serum FABP4 levels.

Variable	B	*β*	95% CI	*p*
Serum FABP4	0.89	0.39	0.15–0.64	**0.002** *
HDL	−0.57	−0.44	−0.69 to −0.19	**0.0011** *
umbilical cord serum ghrelin	−3.14	−0.27	−0.52 to −0.02	**0.037** *

Adjusted for the serum FABP4 levels, the maternal and umbilical cord serum ghrelin levels, the maternal and umbilical cord serum leptin levels, HDL, LDL, triglycerides, and BMI at delivery. Unstandardized *β* coefficients with 95% confidence interval and B linear regression coefficients are shown. Statistically significant values are given in bold. * *p* < 0.05; BMI—body mass index; HDL—high-density lipoprotein cholesterol; FABP4—fatty acid binding protein 4.
